# Ejection Fraction Estimation from Echocardiograms Using Optimal Left Ventricle Feature Extraction Based on Clinical Methods

**DOI:** 10.3390/diagnostics13132155

**Published:** 2023-06-24

**Authors:** Samana Batool, Imtiaz Ahmad Taj, Mubeen Ghafoor

**Affiliations:** 1 Electrical Engineering, Capital University of Science and Technology, Islamabad Expressway, Kahuta Road, Islamabad 44000, Pakistan; samana.batool@gmail.com (S.B.); imtiaztaj@cust.edu.pk (I.A.T.); 2 School of Computer Science, University of Lincoln, Brayford Way, Brayford, Pool, Lincoln LN6 7TS, UK

**Keywords:** medical imaging, transthoracic echocardiography, left ventricle ejection fraction, regression, machine learning, Simpson’s biplane method

## Abstract

Echocardiography is one of the imaging systems most often utilized for assessing heart anatomy and function. Left ventricle ejection fraction (LVEF) is an important clinical variable assessed from echocardiography via the measurement of left ventricle (LV) parameters. Significant inter-observer and intra-observer variability is seen when LVEF is quantified by cardiologists using huge echocardiography data. Machine learning algorithms have the capability to analyze such extensive datasets and identify intricate patterns of structure and function of the heart that highly skilled observers might overlook, hence paving the way for computer-assisted diagnostics in this field. In this study, LV segmentation is performed on echocardiogram data followed by feature extraction from the left ventricle based on clinical methods. The extracted features are then subjected to analysis using both neural networks and traditional machine learning algorithms to estimate the LVEF. The results indicate that employing machine learning techniques on the extracted features from the left ventricle leads to higher accuracy than the utilization of Simpson’s method for estimating the LVEF. The evaluations are performed on a publicly available echocardiogram dataset, EchoNet-Dynamic. The best results are obtained when DeepLab, a convolutional neural network architecture, is used for LV segmentation along with Long Short-Term Memory Networks (LSTM) for the regression of LVEF, obtaining a dice similarity coefficient of 0.92 and a mean absolute error of 5.736%.

## 1. Introduction

According to estimates, cardiovascular diseases (CVDs) account for the majority of fatalities worldwide and approximately claim 18.6 million lives annually, 85% of which are attributed to heart attacks and stroke [1]. Heart disease specifically refers to disorders that have an impact on the structure and function of the heart. The detection of heart disease at an early stage is of utmost importance, as obesity, hypertension, and metabolic disorders such as diabetes are on the rise. A growing proportion of people are receiving heart failure diagnoses, and the proportion is estimated to increase by 46% by 2030 [2]. A delay in diagnosis might result in a poor prognosis, which is frequently linked to permanent pathophysiologic alterations that develop over time. Cardiology has benefited from the application of various imaging techniques, which include Computerized Tomography (CT), Cardiovascular Magnetic Resonance Imaging (CMR), Fundus Photography, Intravascular Ultrasound (IVUS), Echocardiography, and several others. Despite the high imaging quality of CMR and CT, one of the most often utilized imaging systems for assessing heart anatomy and function is echocardiography, mainly due to its accessibility, portability, and cheaper cost as compared to other techniques [3]. These characteristics make it possible to fully utilize echocardiography and apply it for diagnosis, even in low-resource situations. Transthoracic Echocardiography (TTE) is the primary diagnostic approach used to assess both acute and chronic heart failure. It offers immediate and valuable information about the condition of the left ventricle (LV), including its end-systolic and end-diastolic volume, the function of the valve, and the thickness of the wall. Moreover, TTE plays a crucial role in providing insights into the underlying causes or contributing factors of heart failure.

Echocardiography presents challenges that are not straightforward for multiple reasons. Rather than comprising a single still image, an echocardiograph study can consist of a number of videos collected from multiple views. This huge number of multidimensional data generated in each study are difficult to comprehend and hence not fully utilized. Additionally, measurements can differ from one video to another due to beat-to-beat inconsistency and variability resulting from the estimation of a three-dimensional object using two-dimensional images. Acquiring operator-dependent data, device inconsistency, and low image quality further restrict echocardiography. Given the presence of these constraints, it appears that echocardiography could make use of an automated learning technique to aid human interpretation. Artificial intelligence (AI) techniques, which include machine learning (ML) and deep learning (DL), can play an effective role in achieving this task. ML, with its developing applications in cardiac ultrasound, enables the automatic detection of cut planes and heart structures, along with a wide range of computerized measurements.

The measurement of the left ventricular ejection fraction (LVEF) is a key outcome in echocardiography. The LV volume estimates are used to calculate the LVEF, which is used to assess the pumping function of the heart. It represents the percentage of blood pumped out of the left ventricle with each contraction, specifically during the systolic phase of the cardiac cycle. It is a vital factor in the evaluation of risk among patients suffering from heart failure [4]. One way to estimate the LVEF from echocardiogram data is to use a technique called Simpson’s rule, which involves tracing the endocardial border of the left ventricle on multiple frames of the echocardiogram to create a volume–time curve. LVEF is determined by calculating the difference between the end-diastolic volume (EDV) and the end-systolic volume (ESV), and then normalizing this difference by dividing it by the EDV; LVEF = (EDV − ESV)/EDV. This method, however, depends on accurately tracing the endocardial border on each frame of the echocardiogram, which can be difficult and time-consuming, particularly when the endocardial border is poorly defined. Consequently, an important step in achieving high measurement accuracy is obtaining reliable LV segmentation. In addition, the exploitation of temporal information in echocardiogram data can provide more accurate and reproducible estimates of the LVEF, which is an important indicator of cardiac function and is used to diagnose and monitor various heart conditions.

For the measurement of LV volumes and LVEF, amongst the two common techniques used in clinical practice, i.e., the area–length method and Simpson’s biplane method, the latter is the most recommended methodology to evaluate LVEF [3]. There is not much work on the use of Simpson’s method in extracting LV features from segmented chambers. ML techniques can be used to explore the use of the extracted features in conjunction with the clinical methods used by cardiologists in routine practice to estimate LV volumes and LVEF. Zhang et al. [5] used the area–length method to compute LV volumes and LVEF from the segmented images. Their method makes the assumption that the LV is bullet-shaped, but this assumption about the shape of the LV does not always hold [3]. In this work, Simpson’s method is studied for the extraction of structural features of the LV, which leads to the estimation of LVEF.

Below are the main contributions of this work.

- The method proposed in this study is to first perform left ventricle segmentation on A4C view of an echocardiogram and then extract pertinent features based on a clinical technique (Simpson’s method) from segmented images, which is consistent with the workflow of the methods used in clinical settings;
- To estimate the LVEF, machine learning methods are applied to features obtained from the LV, and their efficacy, in this case, is investigated;
- To leverage the temporal information present in the frames of an echocardiogram effectively, Simple Recurrent Neural Networks (RNNs) and Long Short-Term Memory Networks (LSTMs) are employed.

The paper is structured as follows: Section 2 provides an overview of the studies conducted on echocardiographic data, focusing specifically on the segmentation of the left ventricle and estimation of LVEF. In Section 3, the dataset and techniques used along with the proposed method are described in detail. The obtained results are presented in Section 4, followed by a comprehensive discussion and conclusion to summarize the findings and implications of the study.

## 2. Related Work

**Figure 1:**
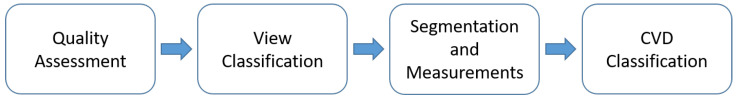
A typical diagnostic system in Automated Echocardiography.

Several studies have focused on the development of automatic interpretation and clinical evaluation systems for medical images [6,7]. An echocardiogram diagnostic system that is based on ML typically comprises automation of different tasks performed by human experts in the clinical investigation, as given in the flow diagram in Figure 1. A considerable amount of work is performed on quality assessment [8,9,10] and view classification [5,11,12,13,14]. A vital component of the automated echocardiography pipeline is the delineation of the left ventricle in order to produce structural and functional indices using the traced boundaries. Automating this procedure of heart chamber segmentation saves time by providing rapid, precise, and objective segmentation throughout the cardiac cycle. To perform segmentation, certain conventional methods for image processing have been proposed, including Otsu thresholding and edge detection [15], a watershed algorithm for LV border segmentation [16] and K-means clustering [17]. These techniques are computationally efficient, but they have a high signal-to-noise ratio and fail to produce acceptable results when there are unclear borders and non-uniform regional intensities. In ML approaches for image segmentation, an image is split into distinct regions by giving each pixel a label of its associated class, as in [5,18,19,20,21,22,23,24,25,26]. Temporal coherence, which is found in heart motion between the frames, is also incorporated along with CNNs by [23] for segmentation.

Other techniques that can be explored for medical image processing and segmentation include [27,28]. Zhang et al. [5] proposed an automated and extensible echocardiography interpreting process. As part of their approach, they performed preprocessing of complete echocardiogram studies, view classification, image segmentation, and detection of the cardiac cycle using CNNs. Using the segmentation output, they estimated LV length, area, volume, and mass, which further led to the calculation of LVEF using the area–length method. The automated algorithms based on this method tend to overestimate the structural estimates, including LVEF, left ventricular volume, and left atrial volume, when compared to the ground truth values. In addition, large deviations could be seen in the result because of outliers. Furthermore, individual frames that are influenced by cardiac chamber foreshortening or variance from abnormal heartbeats, such as untimely ventricular contractions or cardiac arrhythmia, are not selected in manual segmentation by cardiologists, which might be disregarded in the case of an automated pipeline.

A complete automated pipeline that comprises tasks including the segmentation of the left ventricle, calculation of LVEF, and disease classification using DL techniques is proposed by Ouyang et al. [19]. For left ventricle segmentation, it used labels from human experts for weak supervised learning for each frame of the video. After that, a three-dimensional CNN was trained using native echocardiogram recordings to estimate LVEF for each frame with a mean absolute error of 4.1%. Heart failure with a poor LVEF was also identified. The authors made the dataset Echonet-Dynamic publicly available, which was used in this study and comprised around ten thousand echocardiogram recordings. The proposed DL model did not perform well with the data that had incorrect human labeling, low visual quality, or arrhythmias and heart rate fluctuations. As a result, further research in a variety of clinical contexts is needed to overcome these challenges. This work was further extended by using the same DL model to classify local cardiac structures as well as estimate volumetric measures and heart performance metrics [29]. The identification of left ventricular hypertrophy, aberrant size of the left atrial, and the existence of devices such as defibrillators and pacemakers was also performed. When the model was trained to estimate LVEF directly, the findings were more accurate than when it was estimated using predicted volumes. In work by Ghorbani et al. [29], the prediction of EDV and ESV did not give promising results, with low $R^{2}$ resulting in a higher bias in LVEF, which is calculated from EDV and ESV, as per the clinical convention. To properly evaluate cardiac mobility and correlation in cardiac structures, future studies will require improved use of temporal information across frames. ImageNet weights were used for pre-training the model due to a lack of sufficient data for DL training; however, this might not improve the model performance as expected due to the considerable disparity in image characteristics between ImageNet and echocardiography images.

In Asch et al. [30], the estimation of LVEF was performed without measuring LV volumes. Their approach assumed that during the systolic phase, the ventricle contracts simultaneously along its length and diameter, allowing the calculation of LVEF using the contraction coefficients they measured in both directions without measuring the volumes. The proposed ML algorithm was designed to estimate the minimum values of these coefficients at the end of a contraction. Another DL model was used for the classification and segmentation of two-dimensional frames and Doppler in echocardiograms [31]. For the EchoNet-Dynamic, this study assessed only high-quality views (6286) to measure LVEF. Since the authors identified low view quality as a problem in this dataset, they selected images of appropriate image quality for this work. This puts a limitation on the number of images that may be annotated in this study. In another study [32], the authors employed a DL algorithm based on VGG16 on the EchoNet-Dynamic dataset. Their algorithm obtained an MAE of 8.08% for LVEF, with an RMSE of 11.98 and a correlation of 0.98.

Many existing methods for estimating LVEF on a sequence of frames have heavily relied on deep learning techniques. In contrast, our approach follows a step-by-step methodology that involves performing segmentation and then utilizing the relevant information extracted as features to estimate LVEF. This approach not only provides detailed insights into the intermediate steps involved but also remains consistent with the clinical workflow. By explicitly incorporating the segmentation and feature extraction steps, we can better understand and interpret the underlying processes, enhancing the transparency and interpretability of the LVEF estimation process.

In addition, it is rational to align with the clinical workflow and acknowledge that concentrating on features extracted from the end-diastolic and end-systolic frames may be adequate for precise LVEF prediction, without the need to analyze the entire video sequence. These specific frames correspond to the points of maximum and minimum volumes in the left ventricle, capturing crucial information. By extracting essential information from these frames, the model ensures consistency with clinical practices while achieving accurate LVEF estimation.

## 3. Materials and Methods

### 3.1. Dataset

In this study, the EchoNet-Dynamic dataset [19] is used, which contains 10,030 apical 4-chamber (2D B-mode) echocardiography recordings. EchoNet-Dynamic is so far the largest known labeled echocardiography dataset that is publicly available. The average age of the patients is 68 ± 21, and 49 percent of them are female. The training, validation, and testing sets have 7460, 1288, and 1277 patients, respectively. Each video includes a boundary tracing, also known as volume tracing, of the LV border at the end of systolic and diastolic frames, as well as cardiac parameters, including LVEF, EDV, and ESV, which are taken as the ground truth values.

### 3.2. Traditional Machine Learning Techniques

Traditional machine learning techniques for regression that are used in this study to estimate LVEF include Support Vector Regression (SVR), random forest (RF), decision tree (DT), and linear regression (LR). These traditional machine learning methods each have their strengths and weaknesses and are suitable for different types of regression problems. 

#### 3.2.1. Linear Regression

Linear regression (LR) is a basic and widely used regression method that models the relationship between the independent variables and the dependent variable as a linear function. It assumes a linear relationship between the input features and the target variable, with the goal of minimizing the difference between the predicted values and the actual values.

#### 3.2.2. Support Vector Regression

Support Vector Regression (SVR) is a supervised learning algorithm that aims to find the best hyperplane that maximizes the margin between the predicted values and the target variable. It finds a subset of training examples, called support vectors, that are used to define the hyperplane. SVR can handle linear and non-linear relationships using different kernel types, such as linear, polynomial, or radial basis function (RBF) kernels.

#### 3.2.3. Decision Trees

Decision trees (DTs) represent a flowchart-like structure where each internal node represents a feature or attribute and each leaf node represents a predicted value. The decision tree splits the data based on the values of the features, aiming to minimize the variance within each resulting subset. Decision trees are easy to interpret and can handle both numerical and categorical data. However, decision trees can be susceptible to overfitting, meaning they may not generalize well to unseen data and may instead memorize the training data. To mitigate this issue, the maximum depth of the decision tree can be set. If the maximum depth is set too high, the tree can become overly complex and capture noise or irrelevant patterns in the training data, leading to overfitting. In this study, the maximum depth of the decision tree was carefully selected to achieve a balance between capturing important relationships in the data and avoiding overfitting.

#### 3.2.4. Random Forest

Random forest (RF) is an ensemble learning method that combines multiple decision trees to make predictions. It works by creating a set of decision trees using random subsets of the training data and random subsets of features. Each tree in the random forest independently predicts the target variable, and the final prediction is obtained by averaging or voting on the predictions of individual trees. Random forests are known for their ability to handle high-dimensional data along with non-linear relationships and provide feature importance measures.

### 3.3. Neural-Network-Based Techniques

Neural networks are computational models inspired by the human brain. They consist of interconnected nodes called neurons organized into layers. The network receives input signals, processes them using activation functions, and produces output signals. By adjusting the connections between neurons, known as weights, neural networks can learn patterns and make predictions. They are commonly used in machine learning and have been successful in tasks such as classification and regression.

Recurrent Neural Networks(RNN) and Long Short-Term Memory Networks (LSTM) are popular neural network (NN) architectures used in machine learning for regression tasks involving sequential data. These networks are effective in capturing temporal patterns and dependencies in sequential data, making them valuable tools for regression tasks involving time series or other sequential data formats.

#### 3.3.1. RNN

RNNs are designed to handle sequential data by maintaining a hidden state that captures information from previous time steps. This allows them to capture temporal dependencies and model the dynamics of the sequence. In the context of regression, RNNs can learn to predict the next value in a sequence based on the previous values.

#### 3.3.2. LSTM

LSTM is a variant of RNN that addresses the issue of vanishing gradients, which can occur with RNNs. LSTM introduces a memory cell that can retain information over long periods of time, allowing the network to capture dependencies over longer sequences. It achieves this by using gates that control the flow of information into and out of the memory cell. For regression tasks, RNNs and LSTM networks are trained to predict continuous values based on the input sequence. The input sequence is represented as a time series, where each element corresponds to a specific time step. The network processes the sequence iteratively, updating its hidden state and producing an output at each time step. The final output can be used as the regression prediction.

### 3.4. Proposed Method

**Figure 2:**
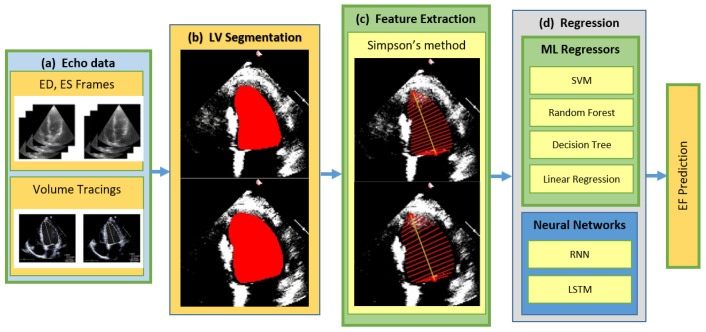
Proposed Method; (**a**) End-Diastolic (ED), End-Systolic (ES) input frames; (**b**) Left Ventricle (LV) segmentation performed with DeepLab; (**c**) Simpson’s diameters extracted from LV; (**d**) Regression performed using ML and NN algorithms.

The proposed method for estimating LVEF from the left ventricle involves two main steps. Firstly, LV segmentation is performed on the A4C views of the dataset using deep learning techniques. The resulting segmented LV masks are then utilized for feature extraction based on Simpson’s method. Regression techniques are subsequently applied to these extracted features in order to estimate LVEF. These techniques encompass both traditional machine learning approaches, chosen for their simplicity and ease of implementation, as described in Section 3.2, as well as neural networks outlined in Section 3.3. The overall framework of the proposed method is illustrated in Figure 2. The different blocks of the framework are described in detail in Section 3.4.1–Section 3.4.3.

Traditional machine learning algorithms present certain advantages. They offer interpretability, require fewer computational resources, and exhibit reliable performance even on smaller datasets with simpler relationships. These algorithms can provide insights and understanding into the underlying patterns within the data. Neural networks, on the other hand, demonstrate their strength in capturing intricate patterns and modeling non-linear relationships. They offer the advantage of requiring less explicit feature engineering and exhibit strong performance on large and complex datasets. Neural networks have showcased exceptional performance across various domains and exhibit good generalization capabilities when appropriately trained, albeit with the caveat of potential overfitting if the model capacity is not effectively controlled. By employing both neural networks and traditional machine learning algorithms, the aim is to leverage the respective strengths of each approach and explore their effectiveness in addressing the research objectives.

#### 3.4.1. LV Segmentation

The input to the LV segmentation module, as shown in Figure 2, consists of the end-diastolic and end-systolic frames. These frames are processed by the LV segmentation module to extract the left ventricle region of interest. In this work, DeepLab is employed for the semantic segmentation of the LV chamber from an echocardiographic image. The use of atrous convolutions, commonly referred to as dilated convolution, is the primary component of the DeepLab model [33]. They enable the model to effectively capture features at different scales. By using the DeepLab model with the ResNet architecture as its backbone, the approach employs atrous convolution in a cascading or parallel manner. This allows for the capturing of multi-scale context by utilizing different atrous rates [33]. Additionally, the inclusion of the Atrous Spatial Pyramid Pooling module, along with image-level features, further enhances the performance of the model.

For the purpose of segmentation training, DeepLabv3, with a Resnet50 backbone, was employed, utilizing the PyTorch framework with pre-trained weights. The model underwent training for a total of 45 epochs, employing a batch size of 16. The SGD optimizer was utilized with an initial learning rate (LR) set to $10^{-3}$. To facilitate learning rate decay, a ’Reduce LR On Plateau’ strategy was employed, decreasing the learning rate when no improvement was observed for a consecutive number of epochs known as the ’patience’. In our case, the ’patience’ value was set to 3.

During the segmentation process, the input image size was standardized to 112 × 112 pixels. The models were trained for A4C views by using the training, validation, and testing sets as mentioned in Section 3.1. The model implementation was carried out using Python version 3.10.10 and PyTorch version 2.0.0, respectively. The experiments were conducted on a server equipped with an NVIDIA Tesla P100 GPU.

#### 3.4.2. Feature Extraction

**Figure 3:**
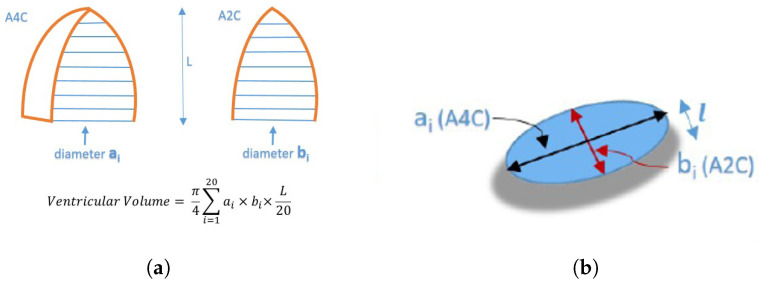
Simpson’s Biplane Method ($a_{i}$—disk diameters (A4C view), $b_{i}$—disk diameters (A2C view), L—length of major axis, *l*—height of a single disk). (**a**) Division of LV into elliptical disks (**b**) A disk in Simpson’s Method.

After LV segmentation, feature extraction is performed on segmented LV based on the modified Simpson’s method. The modified Simpson’s method is the most widely used method in routine clinical practice to find LV volume from the volume tracings [3]. This approach, commonly referred to as the Biplane Method of Disks, divides the left ventricle into a number of elliptical disks aligned perpendicular to the ventricle’s major axis, as shown in Figure 3a. Figure 3b shows a cross-section of one such disk.

The volumeofeachdisk shown in Figure 3b is given by Equation (1):

(1) $$volume\;of\;each\;disk=\frac{\pi (a_{i}\times b_{i})l}{4}$$

where $a_{i}$ and $b_{i}$ are the semi-axis lengths of a disk, obtained from an apical four-chamber and apical two-chamber, respectively, and *l* is the height of the disk. Adding the volumes of these disks gives the total volume of the left ventricle. The respective volumes at the end-systole and end-diastole are used to calculate the LVEF using Equation (2);

(2) $$LVEF=\frac{EDV-ESV}{EDV}\times 100\%.$$

**Figure 4:**
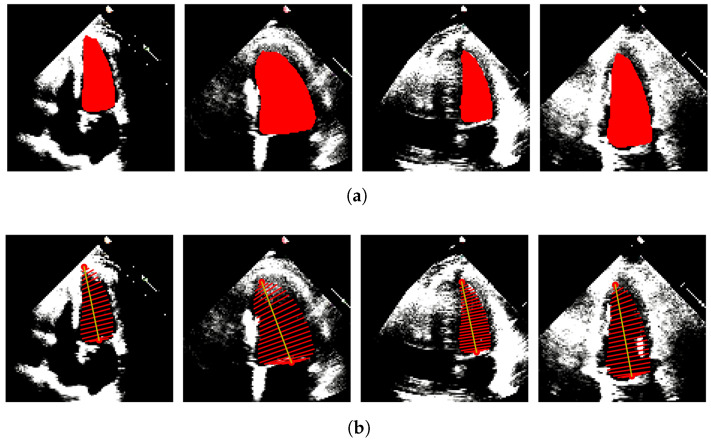
LV Segmentation and Features Extraction on systolic frames. (**a**) LV segmentation masks obtained on systolic frames. (**b**) Diameter tracings obtained on systolic frames.

**Figure 5:**
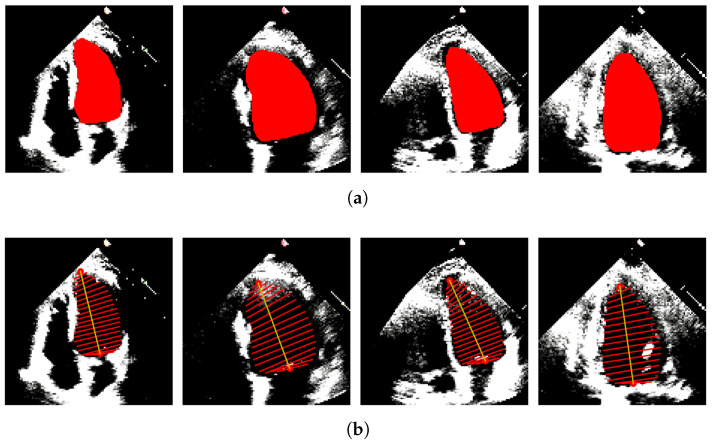
LV Segmentation and Feature Extraction on Diastolic Frames. (**a**) LV segmentation masks obtained on diastolic frames. (**b**) Diameter tracings obtained on diastolic frames.

In our case, only A4C measurements are available; hence, the monoplane Simpson’s method is utilized, which uses measurements obtained from A4C only. To extract features from the segmented images, the contour points (volume tracings) are derived from the segmented mask representing the left ventricle at the end-diastolic and end-systolic frames, respectively. The localization process identifies the mitral valve and apex points, which enable the determination of the major axis of the left ventricle. The major axis is the length between these two points. Subsequently, the disks are positioned orthogonal to the major axis, spaced at the positions obtained by dividing the major axis into 20 equal parts. At each disk position, a region of interest (ROI) is defined as a disk shape centered at that position, and the pixels within this ROI are extracted. To ascertain the diameter of each disk, the maximum distance between any two points within the ROI is computed. Figure 4 and Figure 5 show the segmented images and their corresponding feature extraction at the end-systole and end-diastole, respectively.

#### 3.4.3. Estimation of LVEF

The features extracted from the dataset are utilized as the training set for both machine learning algorithms, discussed in Section 3.2 and neural networks, described in Section 3.3. To evaluate the performance of the model and optimize hyperparameters, a k-fold cross-validation is conducted on a validation set. The trained model is then assessed on a separate test set, employing various metrics, including the correlation coefficient (Corr), mean absolute error (MAE), and root mean squared error (RMSE).

Recognizing the significance of temporal information encompassing the end-systolic and end-diastolic frames in echocardiogram outcomes, RNNs and LSTMs are employed to capture such temporal dependencies and investigate whether this method could yield improved results compared to the traditional machine learning techniques.

The recurrent neural network is a variant of neural networks that is created to effectively handle sequential data. RNNs exhibit remarkable suitability for tasks involving the analysis and generation of sequences, owing to their unique ability to retain internal memory or context. A distinguishing characteristic of RNNs lies in their recurrent connections, which facilitate the transmission of information from one step in the sequence to the subsequent step. This mechanism empowers the network to capture dependencies and discern patterns that unfold over time or sequence. During each time step, the RNN receives an input, which is processed alongside the internal memory derived from the preceding step. The network subsequently produces an output, along with an updated internal memory. This iterative process continues throughout each step in the sequence, establishing a temporal relationship that links the inputs and outputs. The internal memory of an RNN assumes the form of a hidden state, evolving as the network traverses each element of the sequence. This hidden state acts as a repository, preserving pertinent information related to prior inputs and their influence on the current step. The Long Short-Term Memory network is a common version of RNNs. When propagating information over lengthy sequences, ordinary RNNs may have the issue of vanishing gradients. To manage information flow and solve the vanishing gradient issue, LSTMs employ specialized memory cells and gating systems. This makes LSTM a preferred choice when exploiting both spatial and temporal information from the end-systolic and end-diastolic frames.

#### 3.4.4. Hyperparameter Tuning

In this study, the best hyperparameters and the corresponding model were obtained using a grid search with five-fold cross-validation. The hyperparameter values for the ML models, along with their descriptions and the range of values considered for grid search, are provided in Table 1.

Table 2 gives the hyperparameter values for the neural networks. For both simple RNNs and LSTMs, sequential models were constructed using the Keras library, comprising three layers of each followed by a dense layer. To determine the optimal number of hidden units, different configurations were tested. The models’ hyperparameters were fine-tuned to optimize their performance. The models were then compiled using the loss function and optimizer as mentioned for each NN in Table 2. This hyperparameter configuration was chosen based on the goal of minimizing the mean squared error between the predicted and the ground truth values.

## 4. Results

### 4.1. Evaluation Metrics

The dice similarity coefficient (DSC) is used to evaluate the performance of the segmentation tasks by finding the similarity or overlap between the segmented image and the ground truth mask [34,35,36]. It is particularly useful for evaluating the accuracy of binary segmentation masks, as is the case in this study. The formula for calculating the dice similarity coefficient is given in Equation (3).

(3) $$DSC=\frac{2|\hat{\omega }\cap \omega |}{|\hat{\omega }\left|+|\omega \right|}$$

where $\hat{\omega }$ and ω represent the estimate of the segmented mask and the ground truth, respectively. $|\hat{\omega }|$ and |ω| represent the sizes of these masks, and $|\hat{\omega }\left|+|\omega \right|$ represents the intersection of these masks. The DSC ranges from 0 to 1, where 0 indicates no overlap or dissimilarity between the sets, and 1 indicates a perfect match or complete overlap between the sets.

To evaluate how accurately features have been extracted from LV masks based on Simpson’s method, the Hausdorff Distance (HD) and mean absolute error (MAE) between contour points is used. The Hausdorff distance between sets *U* and *V* can be calculated as follows:

(4) $$\left(U,V\right)=\max\left(\max_{u\in U}\min_{v\in V}∥u-v∥,\max_{v\in V}\min_{u\in U}∥u-v∥\right)$$

where *U* represents the set of contour points obtained from the ground truth LV mask and *V* represents the set of contour points obtained from the predicted LV mask. The Hausdorff distance and MAE in this case are given in millimeters (mm).

The accuracy of the regression estimates is measured using mean absolute error (MAE) and root mean squared error (RMSE), which are the percentage errors in the case of LVEF. RMSE gives comparatively large weight to big errors because the errors are squared before being averaged. In our case, undesirably larger errors are present mostly because of a few erroneous files in the data. Hence, MAE in this case is a more desirable metric from an interpretation point of view.

To access the agreement between the estimated LVEF and the ground truth value of LVEF, the Pearson correlation coefficient is used, which is given in Equation (5).

(5) $$Corr=\frac{\sum_{i=1}^{n}\left(p_{i}-\bar{p}\right)\left(q_{i}-\bar{q}\right)}{\sqrt{\sum_{i=1}^{n}{\left(p_{i}-\bar{p}\right)}^{2}\sum_{i=1}^{n}{\left(q_{i}-\bar{q}\right)}^{2}}}$$

where $p_{i}$ and $q_{i}$ are the individual data points and $\bar{p}$ and $\bar{q}$ are the averages of the estimated and ground truth LVEFs, respectively. A perfect correlation has a value of 1, whereas 0 indicates no correlation between *p* and *q*.

### 4.2. Results of LV Segmentation and LVEF Estimation

The overall dice similarity coefficient obtained for LV segmentation is 0.92, which indicates a considerably reasonable similarity index. The DSCs for segmentation of systolic and diastolic frames are given in Table 3. The table also includes the Hausdorff distance and mean absolute error for extracted features (volume tracings) in systolic and diastolic frames.

To estimate LVEF, the set of features derived from Simpson’s method comprises the diameters of the disks obtained from Simpson’s method, along with the length of LV. A combination of regression techniques was applied to this set of features. LSTM and RNN were also used to estimate LVEF. Table 4 shows the results obtained.

We conclude from these results that the best estimations are obtained when LSTM is used for the regression of LVEF on extracted features from LV segmentations. Amongst traditional machine learning methods, SVR and RF also produced comparable results.

**Figure 6:**
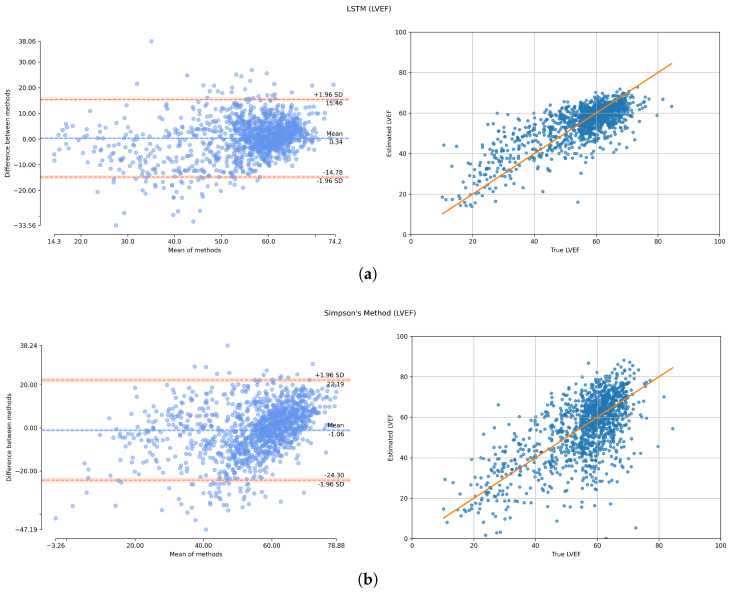
Left: Bland–Altman Plot (The blue line shows the line of perfect average agreement, red lines show limit of agreement bounds at ±1.96 standard deviation) Right: Correlation Plot (Red line shows the line of perfect fit): (**a**) LSTM; (**b**) Simpson’s method.

Figure 6 displays the correlation and Bland–Altman plots, illustrating the relationship between the ground truth and predicted values of LVEF. The comparison is conducted between the utilization of LSTM as a regression technique and the direct application of Simpson’s method for calculating LVEF.

## 5. Discussion

### 5.1. Summary and Contributions

In this study, the proposed method aligns with the clinical workflow of the LVEF estimation and gives insight into the intermediate steps undertaken by cardiologists in the process of LVEF calculation, including LV segmentation and finding volume tracings. By incorporating these essential components, the proposed method not only ensures interpretability but also maintains consistency with the established clinical workflow.

In clinical practice, left ventricle segmentation is typically obtained from the end-diastole and end-systole frames of an echocardiogram. The LV is divided into disks, which are used to calculate the left ventricular end-diastolic and end-systolic volumes. LVEF is then derived using these volumes obtained from both the apical two-chamber (A2C) and apical four-chamber (A4C) views. In this research, the echocardiogram dataset consists solely of A4C views, limiting the use of Simpson’s biplane method, which relies on orthogonal views. To overcome this limitation, machine learning regression algorithms are applied to estimate LVEF using the available A4C data. Additionally, the established clinical workflow is also followed to calculate LVEF according to the equation for the monoplane Simpson’s method for comparison. In situations where orthogonal views, such as A2C and A4C, are not accessible, employing machine learning techniques to estimate LVEF from the diameters of the disks yields improved outcomes.

In this study, both neural networks and traditional machine learning algorithms have been utilized. A key consideration in this research revolves around the intrinsic need to leverage the temporal information embedded within the data. The ejection fraction, which quantifies the difference between the volume of blood pumped into the left ventricle during systole and the volume pumped out during diastole, heavily relies on the temporal information encapsulated between these two consecutive frames. The utilization of recurrent neural network and Long-Short Term Memory models enable the exploitation of this information between systolic and diastolic frames. These network architectures are specifically designed to handle sequential data and capture the temporal dependencies between consecutive inputs, enabling them to learn from the contextual information across time.

The minimum MAE for estimating LVEF was obtained when LSTM was employed for regression with Simpson’s diameters and LV length taken as set of features. In Table 5, a comparative analysis was conducted for LVEF estimation using various existing methods on the EchoNet-Dynamic dataset. The proposed method outperformed the estimates by other studies, demonstrating its superior performance in LVEF estimation. While many studies typically estimate LVEF using many frames extracted from the video sequences, our approach, in which we focused on features extracted solely from the end-diastolic and end-systolic frames, can be sufficient for accurately predicting LVEF, rather than analyzing the entire video sequence. These key frames represent the maximum and minimum volumes of the left ventricle, and by capturing the essential information from these frames, the model can ensure consistency with clinical practices while still achieving accurate LVEF estimation.

There were certain implementation challenges in this study, which include choosing the appropriate machine learning architecture and optimizing its hyperparameters. Extensive experimentation with multiple architectures has been performed, including fully convolutional networks (FCNs) and DeepLab Networks with different combinations of backbone architectures, including ResNet18, ResNet50, and ResNet101, to identify the most suitable segmentation model for our task. Similarly, for the regression model, thorough hyperparameter tuning and cross-validation were conducted to optimize the model’s performance. In addition, deep learning models require substantial computational resources for training and inference. The availability of high-performance computing infrastructure, including GPUs, was essential to efficiently train our models. We acknowledge that the computational requirements may pose challenges in resource-constrained settings, and future work should focus on developing more resource-efficient models without compromising accuracy. Furthermore, there are a few cases in the dataset that contain erroneous volume tracings. The presence of these samples might have decreased the overall efficiency of the results. Some preprocessing steps might be introduced to tackle such cases in the future.

### 5.2. Clinical Relevance and Practical Implications

Estimating LVEF through ML techniques offers several advantages in daily clinical practice. Machine learning algorithms can automate the process of LVEF estimation, reducing the time and effort required by clinicians. This allows for faster and more efficient assessment of cardiac function, enabling timely decision-making and patient management. Moreover, by providing standardized and consistent LVEF measurements, the inter-observer variability commonly seen in manual assessments can be considerably reduced. This ensures greater accuracy and reliability in the evaluation of cardiac function across different healthcare settings and clinicians.

Automated LVEF estimation using ML techniques also has significant applicability in point-of-care ultrasound (POCUS) devices. These devices are typically compact and portable, making them suitable for use in ambulances, remote clinics, and far-flung areas. POCUS devices equipped with ML-based LVEF estimation algorithms can be readily used by non-expert healthcare providers, such as paramedics or clinicians in remote areas and enable real-time LVEF estimation, providing immediate feedback to aid in diagnostic decision-making and patient management. This enables remote consultation, where acquired ultrasound images and LVEF estimations can be shared with experts located in urban centers, facilitating expert guidance.

It is important to note that while these advantages hold great promise, the integration of machine learning into daily clinical practice requires careful validation, standardization, and regulatory considerations. Nonetheless, the potential benefits of machine-learning-based LVEF estimation make it an exciting and promising avenue for enhancing clinical decision-making and patient care.

### 5.3. Limitations and Future Directions

There are several limitations to consider in this work. The performance and generalizability of the proposed method heavily rely on the availability and quality of the training data. Limited access to diverse and representative datasets may impact the model’s ability to generalize to different populations and imaging conditions. The proposed method may also be sensitive to variations in imaging protocols and equipment. Factors such as image resolution, image quality, and anatomical variations among individuals can affect the accuracy and reliability of the LVEF estimation. These limitations provide opportunities for future research and improvements to enhance the accuracy, interpretability, and clinical applicability of the proposed LVEF estimation method.

The current study focused on using the A4C view for LVEF estimation. However, the availability of A2C view data can give an opportunity for further extension of the study. By incorporating the A2C view along with the A4C view already used, it is anticipated that it can potentially improve the accuracy and reliability of the LVEF estimation. Further, machine learning techniques can be used to incorporate patient-specific information and adapt their algorithms to individual characteristics. This allows for a personalized assessment of LVEF, considering factors such as age, gender, comorbidities, and previous medical history. This personalized approach can lead to more tailored treatment plans and improved patient outcomes.

## 6. Conclusions

The objective of this study was to estimate the left ventricle function utilizing echocardiographic videos from the EchoNet-Dynamic dataset, comprising the apical four-chamber (A4C) views. The initial phase entailed the segmentation of the LV from the videos, followed by the extraction of relevant features from the obtained segmentation results. Subsequently, various regression algorithms were employed to estimate the left ventricular ejection fraction based on these extracted features.

Among the regression techniques employed, the LSTM network gave the best results with the least MAE. Additionally, the Support Vector Regression algorithm demonstrated comparable outcomes while offering the advantage of lower computational complexity.

The evaluation of LVEF is limited when relying solely on the apical four-chamber (A4C) view, as its accuracy is heavily dependent on the selection of a single imaging plane. If the chosen plane does not adequately represent the entire LV volume, the measurements may not accurately reflect the true LV function. However, tracing the endocardial border in multiple phases of the cardiac cycle can be a time-consuming and labor-intensive process, especially in challenging scenarios such as poor image quality or fast heart rates. As a result, our proposed techniques offer a valuable alternative by yielding results of acceptable accuracy even when measurements are based on a single imaging plane. This simplifies the analysis process and provides a practical solution for clinical applications.

## Figures and Tables

**Table 1:** Hyperparameters of Machine Learning Algorithms.

ML Algorithm	Hyperparameter	Definition	Range of Values for Grid Search	Values Selected for the Model
SVR *	Kernel type	The kernel function	[Linear, RBF]	RBF
	Degree	Degree of the kernel function	[2–6]	5
	Gamma	Kernel Coefficient	[0.001, 0.01, 0.1, 1]	0.01
	C	Regularization Parameter	[0.1, 1, 10]	10
	Epsilon	Tolerance around the ground truth	[0.1, 0.01, 0.001]	0.1
RF *	n_estimators	Number of trees in the Forest	[100–500]	400
	max_depth	Maximum depth of the tree	[None, 5, 10]	None
	max_features	Number of features to consider when looking for best split	[auto, sqrt, log2]	Auto (40)
DT *	max_depth	Maximum depth of the tree	[5–10]	9
	min_samples_leaf	Minimum number of samples required to be at a leaf node	[5–20]	17
	min_samples_split	Minimum number of samples required to split at internal node	[5–20]	15

* SVR: Support Vector Regression, RF: random forest, DT: decision tree.

**Table 2:** Hyperparameters of neural networks.

Hyperparameter	Simple RNN	LSTM
Model Layers	3	3
Dense Layer	1	1
Nodes in Layers 1, 2 and 3	128, 32, 16 units	64, 32, 16 units
Loss Function	MSE *	MSE *
Optimizer	Adam	SGD *
Batch size	32	32
Epochs	70	100
Learning Rate	0.001	0.001
Activation Function	tanh	tanh

* MSE: Mean Squared Error, SGD: Stochastic Gradient Descent.

**Table 3:** LV Segmentation and Feature Extraction.

	DSC *	HD * (mm)	MAE * (mm)
Segmentation—Systolic	0.930	-	-
Segmentation—Diastolic	0.911	-	-
Volume Tracings—Systolic	-	6.324	6.704
Volume Tracings—Diastolic	-	7.280	5.716

* DSC: Dice Similarity Coefficient, HD: Hausdorff Distance, MAE: Mean Absolute Error.

**Table 4:** Regression on Extracted Features.

Regressors	MAE	RMSE	Corr
LSTM *	5.736	7.726	0.777
Simple RNN *	6.489	9.180	0.746
SVR *	6.727	8.908	0.689
RF *	6.799	9.022	0.677
DT *	6.865	9.084	0.671
LR *	6.736	8.954	0.683
Simpson’s Method	9.492	13.300	0.638

* LSTM: Long short-term memory network, RNN: Simple Recurrent Neural Network, SVR: Support Vector Regression, RF: random forest, DT: decision tree, LR: linear regression.

**Table 5:** Comparative analysis with other studies for LVEF estimation.

Model	MAE	RMSE	Corr
MC3 * [37]	5.91	6.80	-
R2 + 1D * [37]	6.87	7.55	-
LSTM [32]	8.08	11.98	0.348
DL * Based Workflow [31]	6.5	-	0.76
**Proposed Method**	5.73	7.72	0.78

* MC3: Mixed Convolution 3, R2 + 1D: Spatiotemporal Convolutional block, DL: Deep Learning.

## Data Availability

Not applicable.

## Abbreviations

The following abbreviations are used in this manuscript:

LVEF	Left Ventricle Ejection Fraction
EDV	End-Diastolic Volume
ESV	End-Systolic Volume
LSTM	Long Short Term Memory
RNN	Recurrent Neural Network
SVR	Support Vector Regressor
RF	Random Forest
DT	Decision Tree
LR	Linear Regression
HD	Hausdorff Distance
MAE	Mean Absolute Error
RMSE	Root Mean Square Error

## References

[1] Liu M.B. Cardiovascular Diseases. https://www.who.int/news-room/fact-sheets/detail/cardiovascular-diseases-(cvds).

[2] Benjamin E.J., Blaha M.J., Chiuve S.E., Cushman M., Das S.R., Deo R., De Ferranti S.D., Floyd J., Fornage M., Gillespie C. (2017). Heart Disease and Stroke Statistics’2017 Update: A Report from the American Heart Association. Circulation.

[3] Lang R.M., Badano L.P., Mor-Avi V., Afilalo J., Armstrong A., Ernande L., Flachskampf F.A., Foster E., Goldstein S.A., Kuznetsova T. (2015). Recommendations for cardiac chamber quantification by echocardiography in adults: An update from the American society of echocardiography and the European association of cardiovascular imaging. Eur. Heart J. Cardiovasc. Imaging.

[4] Agha S.A., Kalogeropoulos A.P., Shih J., Georgiopoulou V.V., Giamouzis G., Anarado P., Mangalat D., Hussain I., Book W., Laskar S. (2009). Echocardiography and Risk Prediction in Advanced Heart Failure: Incremental Value Over Clinical Markers. J. Card. Fail..

[5] Zhang J., Gajjala S., Agrawal P., Tison G.H., Hallock L.A., Beussink-Nelson L., Lassen M.H., Fan E., Aras M.A., Jordan C.R. (2018). Fully automated echocardiogram interpretation in clinical practice: Feasibility and diagnostic accuracy. Circulation.

[6] Gao Z., Pan X., Shao J., Jiang X., Su Z., Jin K., Ye J. (2022). Automatic interpretation and clinical evaluation for fundus fluorescein angiography images of diabetic retinopathy patients by deep learning. Br. J. Ophthalmol..

[7] Lu S., Yang B., Xiao Y., Liu S., Liu M., Yin L., Zheng W. (2023). Iterative reconstruction of low-dose CT based on differential sparse. Biomed. Signal Process. Control.

[8] Abdi A.H., Luong C., Tsang T., Allan G., Nouranian S., Jue J., Hawley D., Fleming S., Gin K., Swift J. (2017). Automatic Quality Assessment of Echocardiograms Using Convolutional Neural Networks: Feasibility on the Apical Four-Chamber View. IEEE Trans. Med. Imaging.

[9] Abdi A.H., Luong C., Tsang T., Jue J., Gin K., Yeung D., Hawley D., Rohling R., Abolmaesumi P. (2017). Quality assessment of echocardiographic cine using recurrent neural networks: Feasibility on five standard view planes. Lecture Notes in Computer Science (including subseries Lecture Notes in Artificial Intelligence and Lecture Notes in Bioinformatics), Proceedings of the Medical Image Computing and Computer Assisted Intervention—MICCAI 2017, 20th International Conference, Quebec City, QC, Canada, 11–13 September 2017.

[10] Razaak M., Martini M.G. (2016). CUQI: Cardiac ultrasound video quality index. J. Med. Imaging.

[11] Gao X., Li W., Loomes M., Wang L. (2017). A fused deep learning architecture for viewpoint classification of echocardiography. Inf. Fusion.

[12] Madani A., Arnaout R., Mofrad M., Arnaout R. (2017). Fast and accurate classification of echocardiograms using deep learning. NPJ Digit. Med..

[13] Madani A., Ong J.R., Tibrewal A., Mofrad M.R.K. (2018). Deep echocardiography: Data-efficient supervised and semi-supervised deep learning towards automated diagnosis of cardiac disease. NPJ Digit. Med..

[14] Vaseli H., Liao Z., Abdi A.H., Girgis H., Behnami D., Luong C., Taheri Dezaki F., Dhungel N., Rohling R., Gin K. (2019). Designing lightweight deep learning models for echocardiography view classification. Medical Imaging 2019: Image-Guided Procedures, Robotic Interventions, and Modeling.

[15] Santos J., Celorico D., Varandas J., Dias J. Automatic segmentation of echocardiographic left ventricular images by windows adaptive thresholds. Proceedings of the International Congress on Ultrasonics.

[16] MeloJúnior S.A., Macchiavello B., Andrade M.M., Carvalho J.L., Carvalho H.S., Vasconcelos D.F., Berger P.A., da Rocha A.F., Nascimento F.A. (2010). Semi-automatic algorithm for construction of the left ventricular area variation curve over a complete cardiac cycle. BioMed. Eng. Online.

[17] John A., Jayanthi K.B. Extraction of cardiac chambers from echocardiographic images. Proceedings of the 2014 IEEE International Conference on Advanced Communication, Control and Computing Technologies, ICACCCT 2014.

[18] Kim T., Hedayat M., Vaitkus V.V., Belohlavek M., Krishnamurthy V., Borazjani I. (2021). Automatic segmentation of the left ventricle in echocardiographic images using convolutional neural networks. Quant. Imaging Med. Surg..

[19] Ouyang D., He B., Ghorbani A., Yuan N., Ebinger J., Langlotz C.P., Heidenreich P.A., Harrington R.A., Liang D.H., Ashley E.A. (2020). Video-based AI for beat-to-beat assessment of cardiac function. Nature.

[20] Silva J.F., Silva J.M., Guerra A., Matos S., Costa C. Ejection Fraction Classification in Transthoracic Echocardiography Using a Deep Learning Approach. Proceedings of the IEEE Symposium on Computer-Based Medical Systems.

[21] Sirjani N., Moradi S., Oghli M.G., Hosseinsabet A., Alizadehasl A., Yadollahi M., Shiri I., Shabanzadeh A. (2022). Automatic cardiac evaluations using a deep video object segmentation network. Insights Imaging.

[22] Yue Z., Li W., Jing J., Yu J., Yi S., Yan W. Automatic segmentation of the Epicardium and Endocardium using convolutional neural network. Proceedings of the International Conference on Signal Processing Proceedings, ICSP.

[23] Chen Y., Zhang X., Haggerty C.M., Stough J.V. (2021). Assessing the generalizability of temporally coherent echocardiography video segmentation. Medical Imaging 2021: Image Processing.

[24] Siefert A.W., Icenogle D.A., Rabbah J.P.M., Saikrishnan N., Rossignac J., Lerakis S., Yoganathan A.P. (2013). Accuracy of a mitral valve segmentation method using j-splines for real-time 3D echocardiography data. Ann. Biomed. Eng..

[25] Krishnaswamy D., Hareendranathan A.R., Suwatanaviroj T., Boulanger P., Becher H., Noga M., Punithakumar K. (2022). A New Semi-automated Algorithm for Volumetric Segmentation of the Left Ventricle in Temporal 3D Echocardiography Sequences. Cardiovasc. Eng. Technol..

[26] Baroni M., Barletta G. (2004). Contour definition and tracking in cardiac imaging through the integration of knowledge and image evidence. Ann. Biomed. Eng..

[27] Qin X., Ban Y., Wu P., Yang B., Liu S., Yin L., Liu M., Zheng W. (2022). Improved Image Fusion Method Based on Sparse Decomposition. Electronics.

[28] Liu H., Liu M., Li D., Zheng W., Yin L., Wang R. (2022). Recent Advances in Pulse-Coupled Neural Networks with Applications in Image Processing. Electronics.

[29] Ghorbani A., Ouyang D., Abid A., He B., Chen J.H., Harrington R.A., Liang D.H., Ashley E.A., Zou J.Y. (2020). Deep learning interpretation of echocardiograms. NPJ Digit. Med..

[30] Asch F.M., Poilvert N., Abraham T., Jankowski M., Cleve J., Adams M., Romano N., Hong H., Mor-Avi V., Martin R.P. (2019). Automated Echocardiographic Quantification of Left Ventricular Ejection Fraction without Volume Measurements Using a Machine Learning Algorithm Mimicking a Human Expert. Circ. Cardiovasc. Imaging.

[31] Tromp J., Seekings P.J., Hung C.L., Iversen M.B., Frost M.J., Ouwerkerk W., Jiang Z., Eisenhaber F., Goh R.S., Zhao H. (2022). Automated interpretation of systolic and diastolic function on the echocardiogram: A multicohort study. Lancet Digit. Health.

[32] Blaivas M., Blaivas L. (2022). Machine learning algorithm using publicly available echo database for simplified “visual estimation” of left ventricular ejection fraction. World J. Exp. Med..

[33] Chen L.C., Papandreou G., Kokkinos I., Murphy K., Yuille A.L. (2018). Rethinking Atrous Convolution for Semantic Image Segmentation Liang-Chieh. IEEE Trans. Pattern Anal. Mach. Intell..

[34] Sudre C.H., Li W., Vercauteren T., Ourselin S., Jorge Cardoso M. (2017). Generalised dice overlap as a deep learning loss function for highly unbalanced segmentations. Lecture Notes in Computer Science (Including Subseries Lecture Notes in Artificial Intelligence and Lecture Notes in Bioinformatics), Proceedings of the DLMIA 2017, ML-CDS 2017: Deep Learning in Medical Image Analysis and Multimodal Learning for Clinical Decision Support Quebec City, QC, Canada, 14 September 2017.

[35] Ronneberger O., Fischer P., Brox T. (2015). U-net: Convolutional networks for biomedical image segmentation. Lecture Notes in Computer Science (including subseries Lecture Notes in Artificial Intelligence and Lecture Notes in Bioinformatics), Proceedings of the MICCAI 2015: Medical Image Computing and Computer-Assisted Intervention—MICCAI 2015, Munich, Germany, 5–9 October 2015.

[36] Qadri S.F., Lin H., Shen L., Ahmad M., Qadri S., Khan S., Khan M., Zareen S.S., Akbar M.A., Bin Heyat M.B. (2023). CT-Based Automatic Spine Segmentation Using Patch-Based Deep Learning. Int. J. Intell. Syst..

[37] Ouyang D., He B., Ghorbani A., Lungren M.P., Ashley E.A., Liang D.H., Zou J.Y. EchoNet-Dynamic: A Large New Cardiac Motion Video Data Resource for Medical Machine Learning. Proceedings of the 33rd Conference on Neural Information Processing Systems (NeurIPS 2019).

